# A biomimetic hyaluronic acid‐silk fibroin nanofiber scaffold promoting regeneration of transected urothelium

**DOI:** 10.1002/btm2.10268

**Published:** 2021-11-18

**Authors:** Yuqing Niu, Massimiliano Galluzzi, Fuming Deng, Zhang Zhao, Ming Fu, Liang Su, Weitang Sun, Wei Jia, Huimin Xia

**Affiliations:** ^1^ Department of Pediatric Surgery Guangdong Provincial Key Laboratory of Research in Structural Birth Defect Disease, Guangzhou Women and Children's Medical Center, Guangzhou Medical University Guangzhou Guangdong China; ^2^ Materials Interfaces Center, Shenzhen Institutes of Advanced Technology, Chinese Academy of Sciences Shenzhen Guangdong China

**Keywords:** electrospinning, hyaluronic acid, regeneration, silk fibroin, tissue engineering

## Abstract

This study was designed to investigate the regulatory effect of hyaluronic acid (HA)—coating silk fibroin (SF) nanofibers during epithelialization of urinary tract for urethral regeneration. The obtained electrospun biomimetic tubular HA‐SF nanofiber scaffold is composed of a dense inner layer and a porous outer layer in order to mimic adhesion and cavernous layers of the native tissue, respectively. A thin layer of HA‐gel coating was fixed in the inner wall to provide SF nanofibers with a dense and smooth surface nano‐topography and higher hydrophilicity. Compared with pure SF nanofibers, HA‐SF nanofibers significantly promoted the adhesion, growth, and proliferation of primary urothelial cells, and up‐regulate the expression of uroplakin‐3 (terminal differentiation keratin protein in urothelium). Using the New Zealand male rabbit urethral injury model, the scaffold composed of tubular HA‐SF nanofibers could recruit lumen and myoepithelial cells from the adjacent area of the host, rapidly reconstructing the urothelial barrier in the wound area in order to keep the urinary tract unobstructed, thereby promoting luminal epithelialization, smooth muscle bundle structural remodeling, and capillary formation. Overall, the synergistic effects of nano‐topography and biophysical cues in a biomimetic scaffold design for effective endogenous regeneration.

**Abbreviations**
ATR‐FTIR: Fourier transform infrared spectra with attenuated total reflection head
DAPI: diamidino‐2‐phenylindole
DMEM: Dulbecco's modified Eagle's medium
ECM: extracellular matrix
EDC: 1‐ethyl‐3‐[3‐dimethylaminopropyl] carbodiimide hydrochloride
FBS: fetal bovine serum
HA: hyaluronic acid
HFIP: hexafluoroisopropanol
IgG: immunoglobulin‐G
K5: cytokeratin‐5
P/S: penicillin/streptomycin
PBS: phosphate buffered saline
RT: room temperature
SEM: scanning electron microscopy
SF: silk fibroin
SMC: smooth muscle cell
TCPS: tissue culture polystyrene
TGA: thermo‐gravimetric analysis
UC: urothelial cells
VVG: Verhoeff Van‐Gieson
XPS: X‐ray photoelectron spectroscopy
α‐SMA: α‐smooth muscle actin

## 1 INTRODUCTION

Urethral trauma is a frequent and costly injury. It may be caused by a multitude of factors, including physical injury, inflammation, ischemic stenosis, congenital defects, or malignant tumor.^1^, ^2^, ^3^ Urethral injuries can seriously impair bladder functionality by exposing urinary tract epithelial nerve fiber receptors to toxins, resulting in inflammation, a main precursor to voiding dysfunction and chronic interstitial cystitis.^4^, ^5^, ^6^ The standard clinical operation method for urethral reconstruction involves the selection of autograft tissue from penis skin flap or oral mucosa tissue.^6^, ^7^ However, such method has a number of disadvantages, such as donor injury, limited donor tissue leading to multiple subsequent surgeries, and various possible treatment complications, such as urinary fistula, urethral stricture, and fibrotic scar tissue formation.^8^, ^9^ Urethral grafts prepared by traditional tissue‐engineering methods have good performance, high patency, and best biocompatibility,^4^, ^6^, ^8^, ^9^, ^10^ which are consistent with our previous report that cell scaffold is a necessary condition for successful tissue regeneration of tubular structure.^11^, ^12^, ^13^ However, the invasiveness, high cost, and long production time seriously limit their clinical translation.

In situ tissue‐engineering using degradable biomaterials can utilize the regeneration potential of human body through reasonable scaffold design (including structural optimization and functionalization) and reproduce natural tissue regeneration.^14^ In this field, some studies have shown that in situ tissue‐engineered scaffolds produced new urethras nearly free of foreign materials in vivo.^15^, ^16^, ^17^ However, few of them can reproduce the biological function of damaged urethra in the early stage of transplantation, and there are difficulties in subsequent lumen epithelial tissue remodeling.

Urothelium is a stratified transitional epithelium derived from the endoderm, extending from the renal pelvis to the proximal urethra, serving as a key barrier between the blood and urine.^3^ It is composed of cytokeratin‐5 (K5) expressing basal cells, intermediate cells, and surface cells specialized for synthesis and transport of uroplakins that assemble into the apical barrier.^5^, ^18^ The migration of urothelial cells (UCs) usually occurs during urethral regeneration in response to urethral injury and disease.^18^, ^19^, ^20^ It has been found that urethral stem/progenitor cells can differentiate into UCs in vitro and in vivo.^4^, ^9^, ^10^ However, little is known about leveraging the recruitment and migration of mature UCs as a strategy to regenerate the urethral epithelium at the injury zone.

Among the potential migration enhancements for epithelial cells, hyaluronic acid (HA), a critical element of extracellular matrix (ECM),^21^, ^22^ can be used to bind with the cell surface receptors (e.g., CD44, RHAMM, and ICAM‐1)^23^, ^24^, ^25^ or to tune the mechanical properties of the ECM,^26^, ^27^ in turn, promoting cell motility and connecting tissues. With a wealth of enriching features such as high hydrophilicity,^28^ immune neutrality,^29^, ^30^ and biodegradability,^31^ HA and its derivatives have been widely used as suitable materials for surgical implants (e.g., cosmetic surgery) and tissue regeneration,^28^, ^29^, ^30^ or as a drug conjugate for targeted delivery.^32^, ^33^ Previous work in our laboratory has shown that the HA‐coated collagen fiber composite scaffold manufactured by coaxial electrospinning technology can induce regenerative immune response in the traumatic urethral region and can recruit and support the proliferation of urethral stem/progenitor cells.^17^ The results showed that scaffolds mimicking ECM by using protein and HA‐based biomaterials may play a much more important role in guiding the development of cell behavior and the formation of functional tissue than previously thought. However, limited mechanical properties and rapid degradation restrict the use of collagen‐based scaffolds in tubular geometry.^15^, ^16^, ^17^, ^34^

Silk fibroin (SF) is a natural protein with excellent mechanical properties, good cell compatibility, controllable degradation, and versatile process‐ability in different material formats.^35^, ^36^, ^37^ The potential of SF nanofibers in regenerative medicine has been investigated in some tissue‐engineering applications, such as urethras,^5^, ^16^ skin,^38^ tendon,^39^ and bone regeneration.^40^ However, to our knowledge, the utilization of biomimetic urethral scaffolds built with HA and SF nanofibers has not been explored in urethral reconstruction. Herein, HA was integrated on the surface of SF nanofibers by electrospinning technology to prepare biomimetic urethral scaffolds based on biomimetic principles. Degradable SF nanofibers components provide sufficient mechanical strength and appropriate peptide/protein for urethral scaffold. HA coating integrated on the surface of SF fiber imparted glycoproteins to the hybrid scaffold, supporting UCs motility and organization, contributing to epithelialization and physiological function reestablishment. To testify this, the surface topography and physicochemical properties of HA‐SF nanofiber scaffolds were characterized by scanning electron microscopy (SEM), X‐ray photoelectron spectroscopy (XPS), static water contact angle measurement, Fourier transform infrared spectra analysis with attenuated total reflection head (ATR‐FTIR), thermo‐gravimetric analysis (TGA), tensile test and atomic force microscopy (AFM). On this basis, the adhesion, proliferation, and phenotype of primary UCs on HA‐SF nanofibers were studied. Finally, the urethral scaffold composed of tubular HA‐SF nanofibers was constructed and implanted into the defect site of rabbit urethra for in vivo urethral regeneration assessment.

## 2 RESULTS

### 2.1 Characterization of SF and HA‐SF nanofibers

**FIGURE 1:**
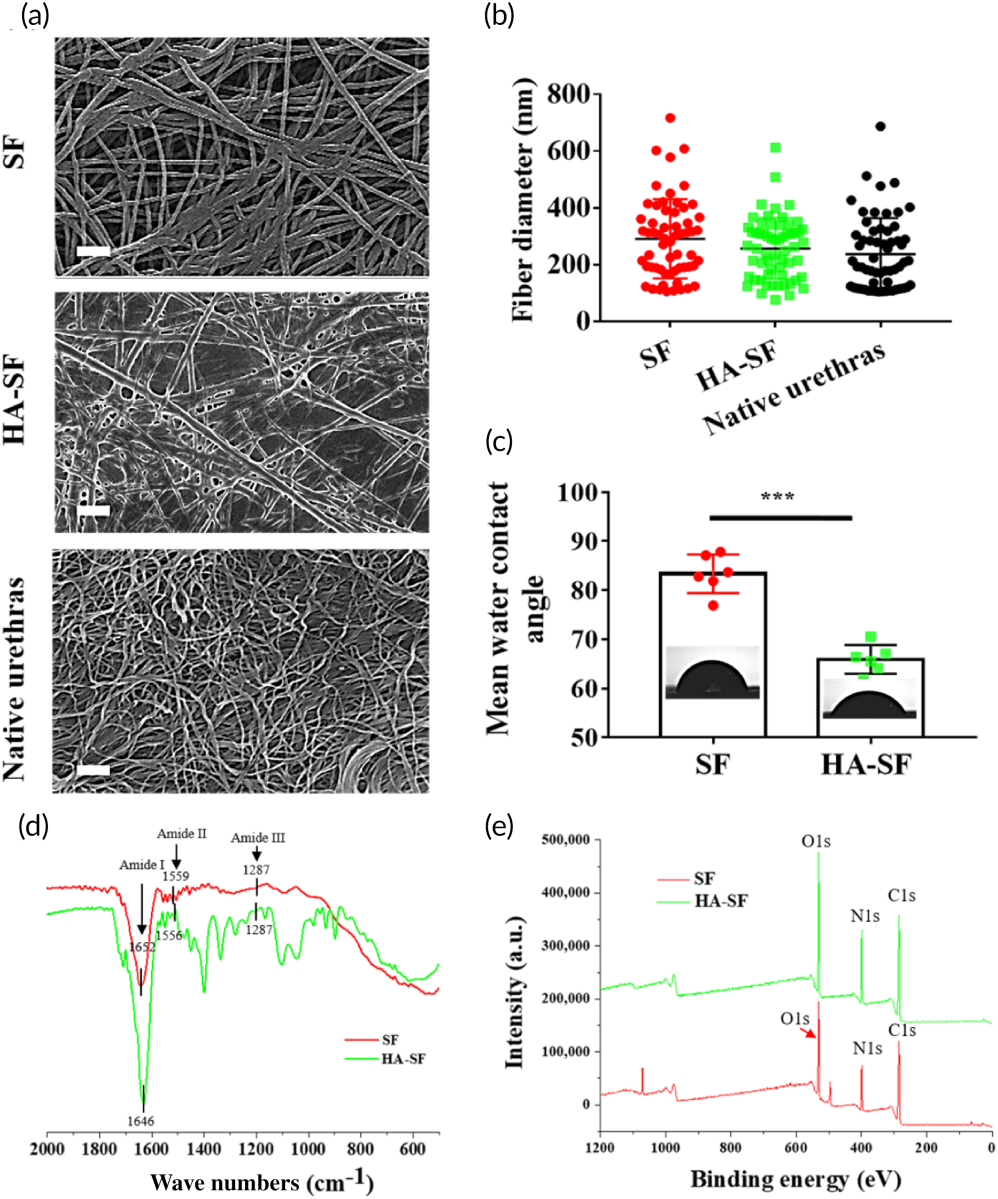
Surface and mechanical characterization of the silk fibroin (SF) and hyaluronic acid (HA)‐SF nanofibers. (a) Representative scanning electron microscopy (SEM) of the inner surface of each tissue‐engineered scaffold and native urethral epithelium (acellular). Scale bars, 1 μm. (b) Statistical data of nanofiber size of the abovementioned tissue‐engineered scaffold and native urethral epithelium (*n* = 60). (c) Mean static water contact angle of the inner surface of each tissue‐engineered scaffold. Inset images are water contact angle photographs of the inner surface of the SF and HA‐SF nanofiber (*n* = 8). (d) Fourier transform infrared spectra analysis with attenuated total reflection (ATR‐FTIR) spectra of a SF and HA‐SF nanofiber films. (e) X‐ray photoelectron spectroscopy (XPS) spectrum of the inner surface SF and HA‐SF nanofiber films. ***p < 0.01

Using electrospinning, the inner wall surface nano‐topography of tubular SF and HA‐SF nanofibers present a smooth and porous nanofiber network, with the average diameters in the range of about (253 ± 15) nm and (222 ± 10) nm, respectively (Figure S1). To increase architecture stability, tubular SF and HA‐SF nanofibers were treated with EDC/ethanol solution. The nano‐topography of tubular SF and HA‐SF inner surface changed significantly after treatment (Figure 1a; Figure S1). The inner surface of SF exhibits tight interlinked nanofiber network nano‐topography, and the HA‐SF interconnect nanofiber network surface is decorated with gel‐like concave coating morphology (Figure 1a). The surface nano‐topography of the HA‐SF nanofibers is very close to the microscopic morphology of the epithelial layer of native urethra. Quantitatively, the mean diameters of SF and HA‐SF nanofibers were in the range of (286.3 ± 16.7) nm and (254 ± 13) nm, respectively, which was slightly larger than that (237.2 ± 16) nm of the native urethral tissue (Figure 1b). Since the inner wall surface of HA‐SF has a layer of HA gel‐like coating, the obtained tubular architecture is composed of a dense inner layer and a porous outer layer, while the SF scaffold is composed of multiple porous layers (Figure S2).

HA is a highly hydrophilic biopolymer, so it is reasonable to expect that the SF nanofibers coated by HA have high wettability, which can be determined by water contact angle measurement. As shown in Figure 1c and Table S1, the average water contact angles of SF and HA‐SF nanofibers are in the range of (80 ± 2)° and (65.9 ± 1)°, respectively. Suggested that HA‐coating can improve the hydrophilicity of SF nanofibers, probably due to the presence of hydroxyl and carboxyl groups in HA molecules.

The chemical distribution of HA‐SF nanofibers was determined by ATR‐FTIR (Figure 1d). In the ATR‐FTIR spectrum, the characteristic absorption peaks of SF nanofibers are obvious at 1652, 1559, and 1287 cm^−1^, consistent with that of amide I, amide II, and amide III, respectively. Compared with SF nanofiber film, the ATR‐FTIR spectra of HA‐SF nanofiber show obvious additional absorption peaks at 1646, 1556, and 1287 cm^−1^. The band shifts of amide I and II were observed. Other studies have found similar band shifts.^37^, ^38^, ^39^, ^40^ These changes may be due to the strong interaction between SF and HA molecules, such as hydrogen bond and electrostatic interaction, ultimately indicating HA had successfully coated on SF nanofiber. In addition, XPS was used to further confirm that the presence of HA coating had no effect on the surface chemical elements of SF nanofiber (Figure 1e; Figure S3). There are no additional element peaks in the surface chemistry of SF and HA‐SF nanofiber, indicating that this coating method has no obvious effect on the surface chemical element of SF and HA. The existence of HA‐coating was further confirmed by TGA (Figure S4). Compared with HA and SF nanofiber, the T_d10_ of HA‐SF nanofiber is 366.32°C, which is between that of HA and SF nanofibers (Figure S4 and Table S1), further confirms the existence of HA‐coating.

Both SF and HA‐SF nanofibers were tested in tensile mode to generate stress–strain curves and derive tensile properties (Figure S5 and Table S1). The Young's modulus and ultimate tensile strength of HA‐SF nanofibers in dry state are (0.83 ± 0.4) MPa and (1.7 ± 0.4) MPa, respectively, which are close to those of SF nanofibers. However, the elongation at break of HA‐SF is (387 ± 17)%, which is slightly lower than that of SF nanofibers (407 ± 20)%. These tests showed that HA‐SF and SF nanofibers are soft but tough substrate. Moreover, the Young's modulus of HA‐SF and SF nanofibers from AFM indentation test in wet state is (0.82 ± 0.16) and (1.2 ± 0.15) MPa, respectively (Figure S6), which is very close to that of HA‐SF and SF nanofibers in dry state. The Young's modulus of HA‐SF and SF nanofibers is higher than that of natural urethra (~0.3 MPa).^41^ Such mechanical properties highlight the suitability for tissue engineering urethral scaffold transplantation. Moreover, AFM morphology showed that the root mean square roughness of HA/SF nanofibers in water was 425.3 nm (Figure S6a), whereas the root mean square roughness for SF (Figure S6b) is 364.5 nm. The above results show that SF and HA‐SF nanofibers have similar mechanical properties, but the surface properties such as nano‐topography, surface chemistry, and wettability of the inner wall of the scaffolds are quite different.

### 2.2 Primary UCs adhesion, phenotypic expression, and proliferation on nanofiber thin film

**FIGURE 2:**
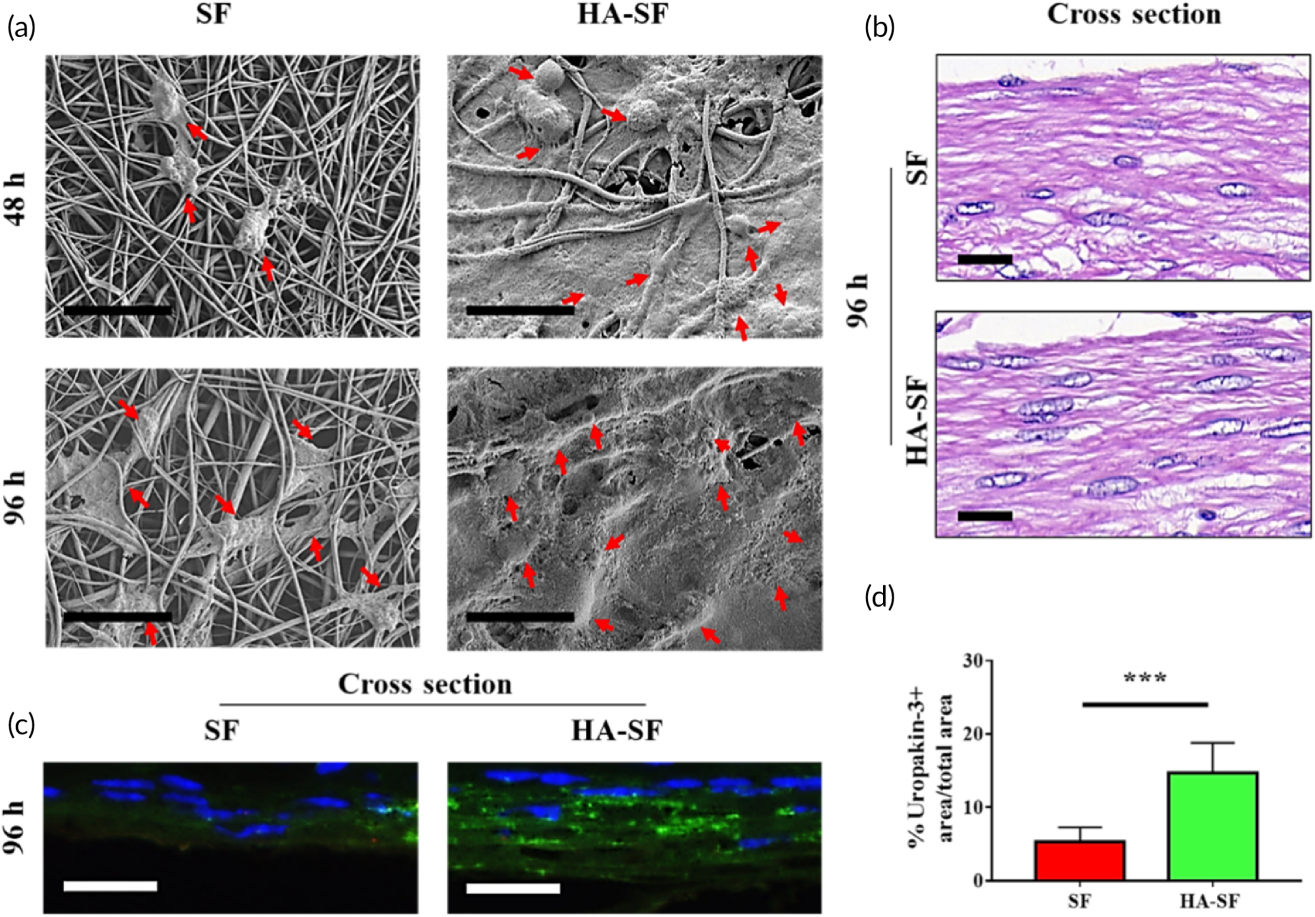
Cell behavior on silk fibroin (SF) and hyaluronic acid (HA)‐SF nanofiber substrates. (a) Scanning electron microscopy (SEM) micrography of primary urothelial cells (UCs) on the inner wall surface of SF and HA‐SF nanofiber after 48 and 96 h postseeding. Red arrows indicate well‐spread UCs. (b) Hematoxylin and eosin (H&E) staining of the cross section of cellularized SF and HA‐SF nanofiber thin films. (c) Fluorescence staining of the cross section of cellularized SF and HA‐SF nanofiber thin films using uropakin‐3 (green) and nuclei (blue). Scale bars: 20 μm (a–c). (d) Statistical data of uropakin‐3 positive expression of primary urethral UCs seeded on different nanofibers (three random fields per sample, *n* = 4 samples per group). ***p < 0.01

We studied the primary UCs growth behavior on the surface of SF and HA‐SF nanofibers. Figure 2a shows typical SEM images of UC on SF and HA‐SF nanofibers at 48 and 96 h after cell seeding. At 48 h, UCs had obvious preferential adhesion on the surface of HA‐SF nanofibers. At 96 h, the adhesion and extension (elongation) of UCs on the surface of HA‐SF nanofibers were more significant. These phenomena were confirmed by observing the cross‐sections of SF and HA‐SF nanofiber films (~80 μm thickness) with hematoxylin and eosin (H&E) staining (Figure 2b; Figure S7). It is worth noting that compared with SF nanofiber thin films, the thin HA gel coating of HA‐SF nanofibers not only contributes to the uniform distribution of UCs on its surface but also its porous SF nanofibers contribute to the ingrowth of UCs.

Meanwhile, uropakin‐3 was used to visualize urothelial plaque in UC to evaluate waterproof and anti‐injury functions. Figure 2c shows the typical confocal laser scanning microscope (CLSM) images of UC uropakin‐3 positive in the cross section of different nanofiber films. The density of uropakin‐3 positive (green fluorescence) on HA‐SF nanofibers was significantly higher than that of SF nanofibers (Figure 2d). These uropakin‐3 staining suggests that HA‐SF nanofibers facilitates the urothelial barrier restoration.

**FIGURE 3:**
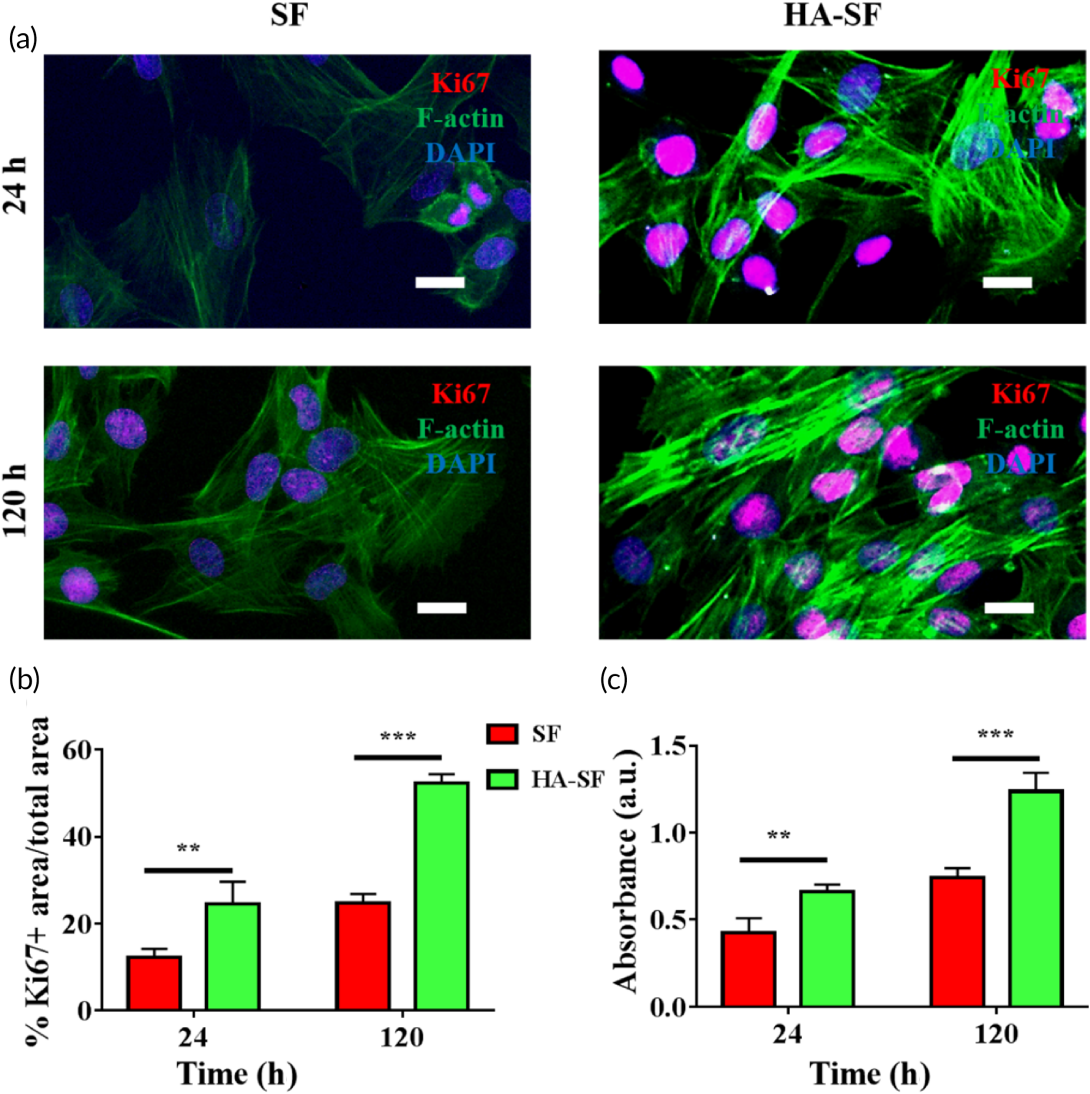
The hyaluronic acid (HA)‐silk fibroin (SF) nanofiber substrates enhancing the proliferation of urothelial cells (UCs). (a) Confocal laser scanning microscope (CLSM) micrographs of stained UCs on the SF and HA‐SF nanofiber substrates after 24 and 120 h of culture. Ki67 (red), cytoskeleton (green), and nuclei (blue). Scale bars, 20 μm. (b) Statistical analysis of the percentage of the proliferation marker Ki67 on each nanofiber surface after 24 and 120 h post seeding (10 random fields per sample, *n* = 4 samples per group). (c) The proliferation of UCs on the surface of SF and HA‐SF at the 24 and 120 h post seeding (10 random fields per sample, *n* = 4 samples per group). **p < 0.01; ***p < 0.01

To study the effects of SF and HA‐SF nanofibers on the proliferation of UCs, we performed immunofluorescence staining of Ki67, a recognized proliferation marker located in the nucleus. In Figure 3a, Ki67 positive cells expressing red fluorescence were observed on the surface of SF and HA‐SF nanofibers at 24 h after cell seeding. By calculating and comparing the levels of Ki67 positive cells, we found that the proliferation of cells on the surface of SF nanofibers was less than that of the HA‐SF nanofibers. At 120 h, a large number of Ki67 positive cells expressing red fluorescence were found on the surface of SF and HA‐SF nanofibers. Quantitatively, the cell proliferation density on SF nanofibers was lower than that of HA‐SF nanofibers (Figure 3b). This result was further confirmed using the cell counting kit‐8 (CCK‐8) method (Figure 3c), showing UCs have good compatibility and can proliferate well on HA‐SF nanofiber surface in vitro. These results clearly indicate that the hydrophilic biomimetic morphology surface of HA‐SF nanofibers may be an ideal substrate for the growth, proliferation, and morphogenesis of primary UCs.

### 2.3 Tubular HA‐SF nanofiber scaffolds accelerated in vivo epithelialization process

**FIGURE 4:**
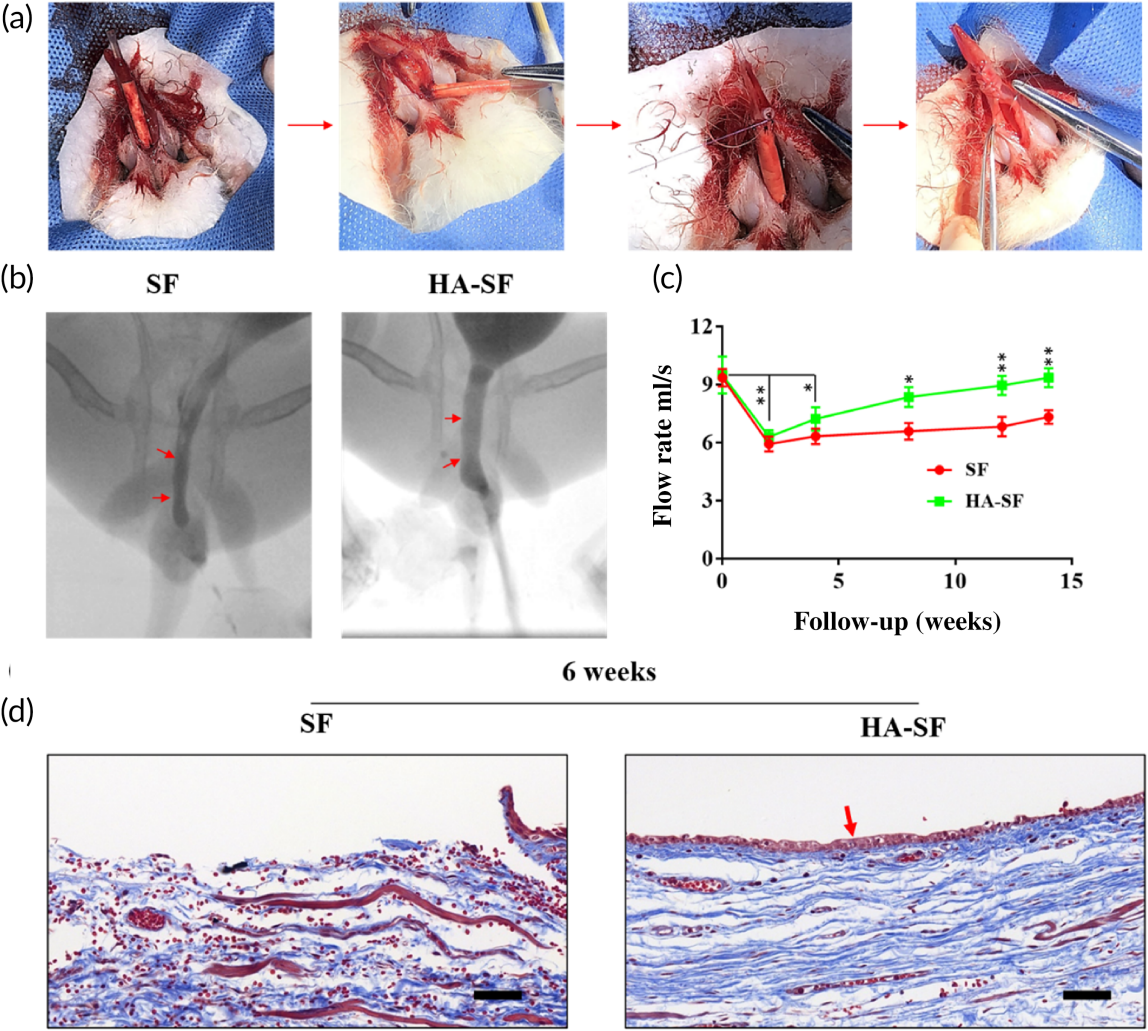
In vivo transplantation process and follow‐up evaluation. (a) The procedure of a tubular hyaluronic acid (HA)‐silk fibroin (SF) graft sutured at the urethral margins in New Zealand rabbits. (b) Voiding cystourethrograms of New Zealand rabbits 12 weeks after operation (red arrows show margins of tissue‐engineered urethras). (c) Uroflow analyses (*n* = 3 animals per group). *p < 0.05; **p < 0.01. (d) Masson's trichrome staining of the cross section of the tubular SF and HA‐SF nanofibers implanted for 6 weeks. Collagen (blue), smooth muscle, and erythrocytes (red). Red arrow indicates that the captured host endogenous urothelial cell (UC) is evenly distributed in the lumen of HA‐SF scaffold. Scale bars, 20 μm

Tubular SF and HA‐SF nanofibers were both applied to a New Zealand rabbit model of iatrogenic urethral trauma (a common urethral defect disease) in order to determine their effect on the process of urethral reconstruction in vivo. We transected New Zealand rabbit urethras and bridged the resulting urethras gaps with tubular SF and HA‐SF nanofibers, respectively (Figure 4a). X‐ray urethrography showed that all grafts maintained patency with the adjacent area of the host native urethra after implantation, and there was no complication of urinary leakage (Figure 4b). By Week 2, all grafted animals had restored partial spontaneous urination function (Figure 4c). By Week 8, the mean urine flow rate of HA‐SF group was (8.4 ± 0.2) ml/s was slightly lower than that (8.9 ± 0.2) ml/s of pre‐implants, significantly higher than that (6.6 ± 0.2) ml/s of SF graft. Furthermore, Masson's trichrome stain was used to histologically examine neo‐tissue remodeling in the regenerated urethral tissue. As shown in Figure 4d, a tight UC layer was formed on the inner wall of HA‐SF scaffold, and these UCs synthesize and deposit nascent collagen along the scaffold. However, the inner wall of SF scaffold did not form a tight UC layer, and the newly formed collagen was disorderly arranged on the scaffold.

**FIGURE 5:**
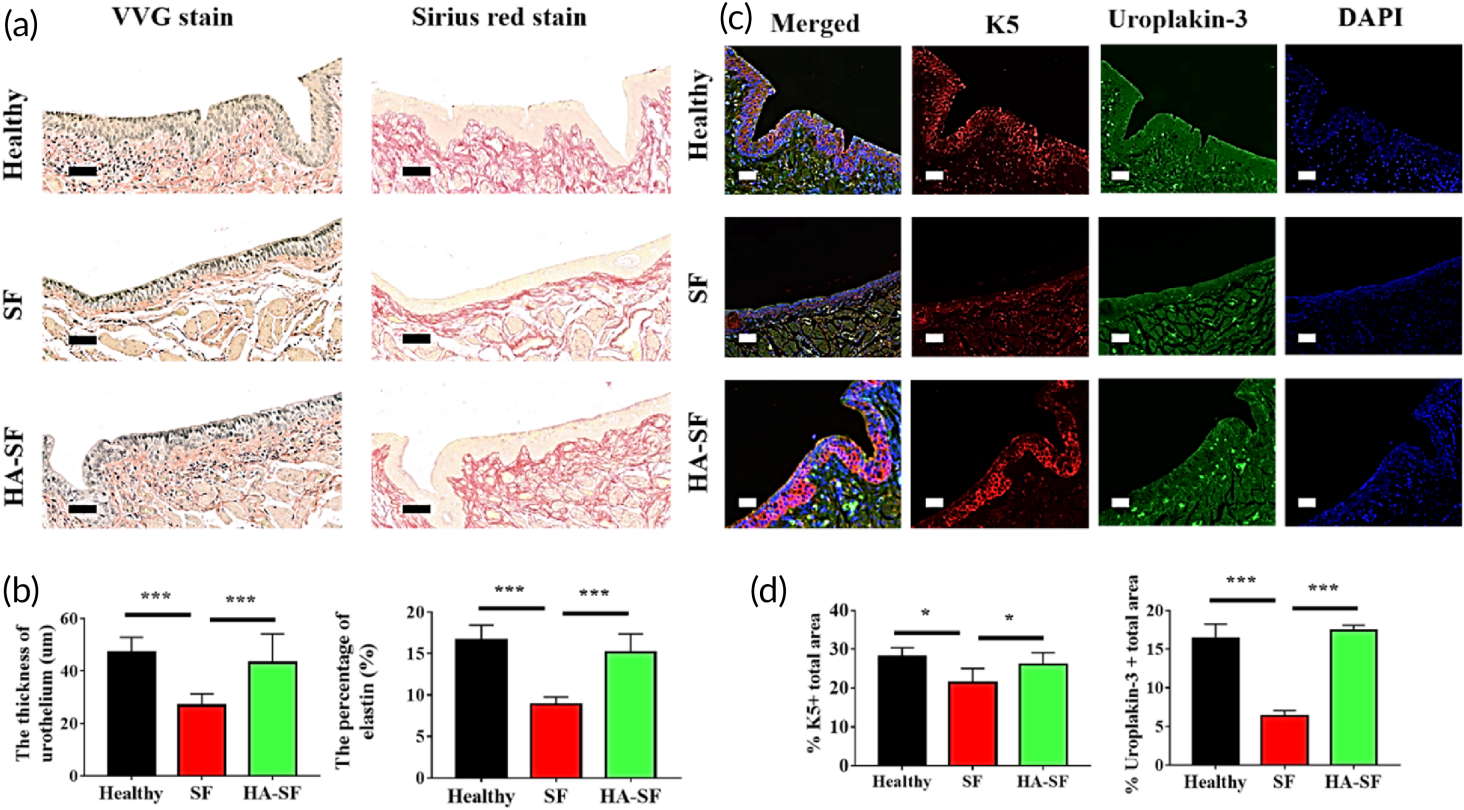
Tubular hyaluronic acid (HA)‐silk fibroin (SF) nanofibers accelerating the repair of urethral epithelium at the site of urethral trauma after 14 weeks transplantation. (a) Verhoeff Van‐Gieson (VVG) and Sirius red staining were used to identify elastin (black) content and epithelial layer (yellow) morphology, respectively. (b) Statistical data of the percentage of elastin nanofibers and the average thickness of epithelial layer in panel a (10 random fields per animal, *n* = 3 animals in each group). (c) Representative confocal laser scanning microscope (CLSM) images of the regenerated urethra epithelium. Blue indicates diamidino‐2‐phenylindole (DAPI) staining positive, magenta staining positive for basal layer progenitor cells, and green indicates positive staining for urethral plaque protein. (d) Statistical of the percentages of K5 positive and Uroplakin‐3 positive in panel c (10 random fields per animal, *n* = 3 animals in each group). Scale bars: (a) 50 μm, (c) 40 μm. *p < 0.05; ***p < 0.01

Verhoeff Van‐Gieson (VVG) and Sirius red staining were performed to observe the urothelial regeneration at 14 weeks after operation (Figure 5a). VVG and Sirius red staining showed that the normal urinary tract epithelium (yellow) was typically stratified, divided into basal layer, middle layer, and surface layer. The cells in the basal layer were monolayer cubic or low columnar, the cells in the middle layer were oval, pear‐shaped, or polygonal, and the cells in the surface layer were oblate. Elastin, which stains as wavy black fiber structures with VVG, is generally distributed in the submucosa and intramuscular layers. VVG and Sirius red staining showed that collagen fibers (red) are present throughout the entire structure, and the curly and wavy collagen fibers were located in the organized bundles of the outer membrane layer. By Week 14, the regenerative urothelial morphology in the SF and HA‐SF groups had also been remodeled into stratified epithelium, with their elastin and collagen fibers were also distributed in the corresponding positions. However, the middle layer and the surface layer of regenerative urothelial epithelium in HA‐SF group were thicker than those in SF group. Quantitatively, the thickness of regenerating urothelium in HA‐SF group was (43 ± 3) μm, significantly thicker than (26 ± 1) μm in SF group and close to the thickness of normal urothelial epithelium (Figure 5b). At the same time, the elastin content in the regenerated urinary tract of HA‐SF group was close to that of normal urinary tract, and was significantly higher than that of SF group (Figure 5b). To further confirm that HA‐SF grafts can promote the remodeling efficiency of hematuria barrier of urothelial, double fluorescent staining of K5 and uroplakin‐3 were carried out in each group. As shown in Figure 5c, at 14 weeks after implantation, there was a layer of K5 positive cuboidal cells in the basal layer of the regenerative urothelial epithelium in the HA‐SF group, and a thin uroplakin‐3 positive flat cell layer at the outermost layer, consistently with the healthy urinary tract epithelium. However, the neo‐urothelium in the SF group was found to show less K5 and uroplakin‐3 expression, which is consistent with the quantitative analysis results (Figure 5d). These findings suggested that urethral grafts made of tubular HA‐SF nanofibers can promote in situ remodeling of the urothelial barrier at the injured site.

### 2.4 HA‐SF scaffold promoted structural remodeling of muscle bundles and blood vessels in the defect area

**FIGURE 6:**
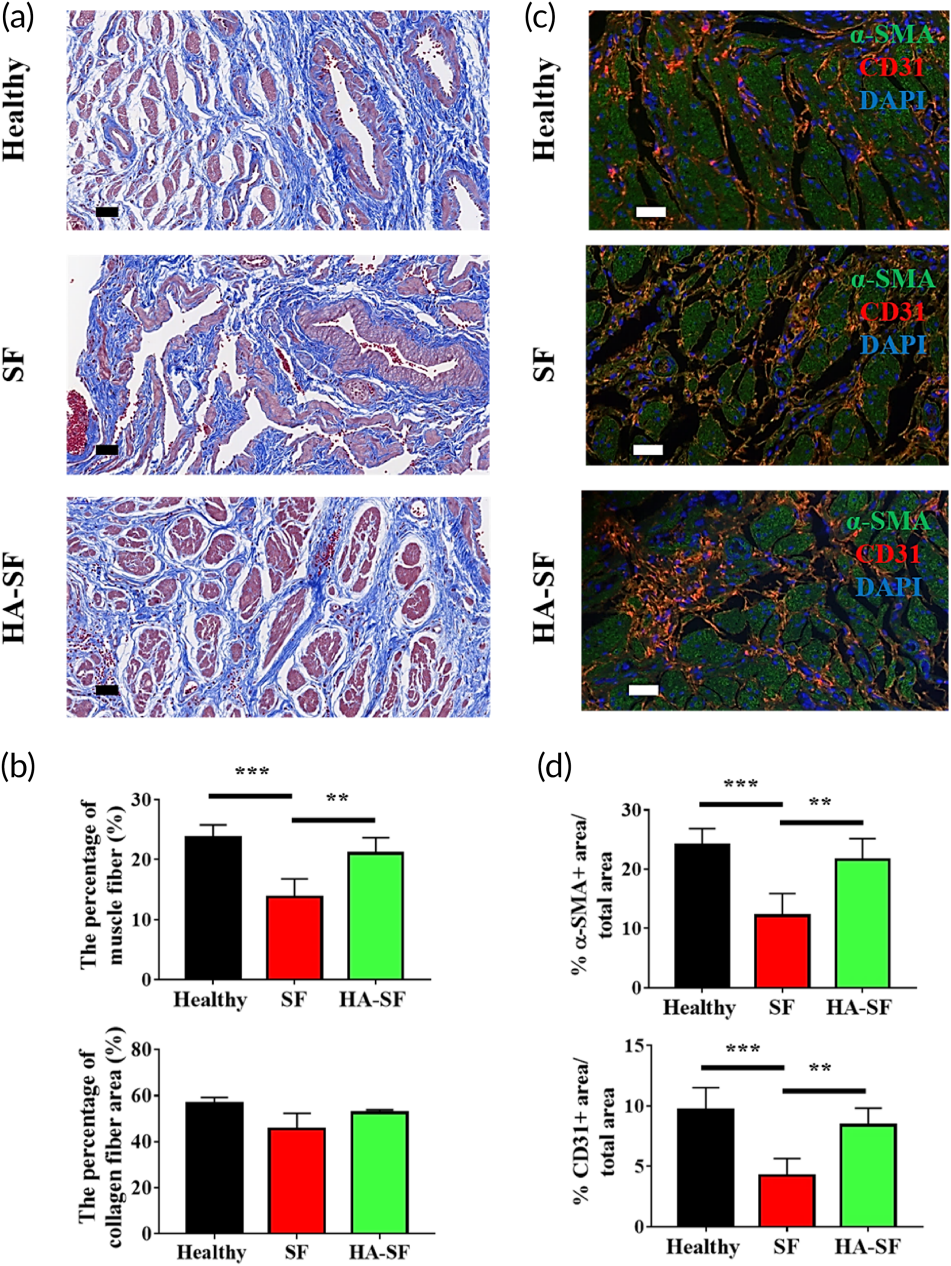
Histological analysis of urethral smooth muscle regeneration 14 weeks after transplantation. (a) Masson's trichrome staining was used to identify collagen fiber (blue) and muscle (red) content. Scale bars: 50 μm. (b) Statistical data of the percentage of collagen nanofibers and muscle in a panel (10 random fields per animal, *n* = 3 animals in each group). (c) Representative confocal laser scanning microscope (CLSM) images of the regenerated urethral smooth muscle and blood vessels at the cross‐section of regenerated urethras. Blue indicates diamidino‐2‐phenylindole (DAPI) staining positive, green staining positive for smooth muscle cells, and red indicates positive staining for blood vessels. Scale bars: 50 μm. (d) Statistical of the percentages of α‐SMA positive and CD31 positive from panel c (10 random fields per animal, *n* = 3 animals in each group). ** *p* < 0.01; *** *p* < 0.01

Next, Masson's trichrome staining was performed to evaluate the regeneration effect of the smooth muscle bundle and collagen nanofibers in the regenerated urethra (Figure 6a). At 14 weeks after implantation, compared with SF graft group, the morphology of newly formed smooth muscle bundle (red) in the regenerated urethra of HA‐SF graft group was close to that of normal urethral than that of SF graft group (Figure 6a,b). The percentage of smooth muscle in the HA‐SF group was (21 ± )%, which was higher than that in the SF group (13.8 ± 1.6)%, and close to (23.7 ± 0.9)% of the normal urethra. The outer layer was composed of smooth, wavy collagen fibers, quantitatively showing no significant difference in the content of collagen fibers in all samples. In addition, immunofluorescence staining confirmed that more α‐SMA positive (green fluorescence) signal was observed in the regeneration zone of HA‐SF graft group, which was close to the α‐SMA positive area of normal urinary tract smooth muscle cells; comparatively, α‐SMA positive signal in the regeneration region of SF graft was weak (Figure 6c,d). The percentage of α‐SMA positive area in HA‐SF graft regeneration area was (21.76 ± 1.6)%, which was close to that (24 ± 1.2)% of normal urethra (*p* > 0.05), and significantly higher than that (12 ± 1.2)% of SF graft group. These results suggest that HA‐SF grafts can induce structural remodeling of smooth muscle cells, rather than recruitment of single cells. As the outer layer of tubular HA‐SF nanofibers is mainly SF nanofibers, its biophysical clues and surface properties are exactly the same as those of pure SF nanofibers. Therefore, we speculate that the urinary epithelium on the inner wall of tubular HA‐SF graft may determine the tissue remodeling pattern of SMC on its outer wall. The formation of vascular network in regenerated tissue is the key to tissue integration.^42^, ^43^ Compared with SF group, there was extensive neovascularization in HA‐SF group (Figure 6c). As shown in Figure 6c,d, there was a strong CD31 positive signal in the regenerated urethra of HA‐SF group, which was evenly distributed in the collagen fibers around the smooth muscle bundle. The CD31 positive region accounted for (8.4 ± 0.6)%, which was close to the vascular density of healthy urethra group. However, the signal of CD31 positive in SF group was weak and scattered sparsely in the collagen fibers around the smooth muscle bundle (Figure 6d). In the process of urethral smooth muscle tissue remodeling, urothelium acts as a barrier on the inner surface of tubular HA‐SF nanofibers to prevent urine from leaking into the underlying tissue.

## 3 DISCUSSION

In this study, we developed a biomimetic tubular nanofiber scaffold that promotes urethral functional recovery by recruiting healing‐associated endogenous UCs to reconstruct the urinary tract epithelial barrier. We found that by precisely controlling the biophysical and biochemical properties of biomaterial scaffolds, endogenous urethral cells can be guided to the defect site to achieve tissue regeneration. This method harnesses the regenerative potential of the human body and eliminates the need for ex vivo cell manipulation.^14^, ^15^

In addition to the limited number of endogenous stem cells and progenitor cells in cartilage, heart, and nerve tissues, in situ tissue engineering provides temporary solutions and alternatives for most of the tissue regeneration.^14^ In situ tissue engineering utilizes bioresponsive scaffolds that harness the inherent regeneration ability of the body. These scaffolds are loaded with biochemical and biophysical clues to recruit endogenous cells for tissue healing. As alternatives to autografts, artificial urethra made of natural degradable biomaterials (such as collagen and SF) are promising candidates in terms of tissue integration,^5^, ^15^, ^16^, ^17^ but lack the capability to induce tissue regeneration due to the bio‐inert characteristics. Hence, the manufacture process of bioactive or bio‐functional urethral grafts has been widely explored through the application of surface modifications and the addition of bioactive molecules. The ECM plays an important role in regulating organogenesis, growth, function, and many human diseases.^23^ As one of the key components of ECM, HA is very important for the migration, proliferation, differentiation, and intercellular communication of many cell types.^24^, ^25^

Herein, urethral graft based on HA and SF with fibrous structure was successfully prepared by sequential electrospinning. The biomimetic nano‐topography on the inner wall surface of tubular HA‐SF nanofibers is enhanced by crosslinking process. By adjusting the size of the receiving device (mold), it is convenient to realize the specific needs of patients for size and diameter. The tubular scaffold was composed of a dense inner layer and a porous outer layer. Its structure simulated the submucosa and cavernous layer of native urethral tissue, respectively. The nano‐topography of the inner wall surface of the crosslinked HA‐SF nanofibers mimics the native urethral epithelium. The main advantage of this structural design is that it successfully combines the protein with polysaccharide, which mimic the key physiological features of ECM.^44^ In vitro cell scaffold co‐culturing experiments showed that the gel‐like coating on the inner wall of HA‐SF scaffold was beneficial to the adhesion of primary UCs on its wall surface, and its hydrophilicity can provide a moisture‐rich micro‐environment for the growth of primary UCs. The interconnected pore networks facilitate UCs infiltration, migration, and scaffold remodeling. The proliferation ability of urethral UCs on HA‐SF nanofibers is higher than that on SF nanofibers, which may be attributed to the bioactivity of HA coating in SF nanofibers. It has been reported that the combination of HA and CD44 can enhance various physiological properties, such as cell matrix adhesion and migration.^23^, ^24^, ^25^ Moreover, the factors that affect the reaction of urethral UCs cells may include matrix stiffness and elasticity.^8^ Here, the Young's modulus of HA‐SF and SF nanofibers measured by our tensile test and AFM shows that HA coating has little effect on the stiffness of matrix. Therefore, the mechanical properties of SF nanofiber scaffold are maintained and matching the native urethral tissue modulus, helping the proliferation of UCs.

One of the advantages of biomimetic tubular HA‐SF nanofiber scaffold is the exploitation of regenerative potential of organism, controlling cell function to realize in situ tissue reconstruction. Moreover, the use of endogenous UCs for in situ urethral regeneration can also reduce immune rejection of transplanted (exogenous) cells, one of the main risks associated with cell therapy.^15^, ^17^ In vivo experiments testify that the biomimetic tubular HA‐SF nanofiber scaffold could direct endogenous UCs to the injury site aiding lumen re‐epithelization in vivo. In this process, the tubular HA‐SF nanofibers provide a structural framework to facilitate the attachment and migration of host endogenous UCs along its inner wall surface. In situ degradation of scaffold is desired for tissue regeneration.^14^, ^15^, ^17^ After implantation to injury site, the in situ degradation of scaffolding architecture of SF and HA‐SF facilitates endogenous‐cell infiltration and scaffold remodeling. Masson's trichrome staining confirmed these phenomena. The monolayer of UCs was formed at 6 weeks after implantation. This lined UCs layer favors the graft to reconstruct the urothelial barrier at the early stage of implantation, thus restoring most of the voiding function. By Week 14, we found that a stratified urothelial epithelium with positive staining of urethral‐specific K5 and uroplakin‐3 was formed in the regenerated urethra. It should be noted that the reconstruction of the urothelial barrier and the structural remodeling of the urethral smooth muscle tract are simultaneous with regeneration effect. Uroflowmetry showed that the mean flow rate in the regenerated urethra was close to the normal range. Because of the low efficiency of the reconstruction of the urinary tract epithelial barrier on the inner wall of the tubular SF scaffold, the structural remodeling process of the urethral smooth muscle bundle and the recovery rate of urinary function is affected. In addition, we also found that the infiltration density of vascular endothelial cells in the regenerated urethra of HA‐SF graft was higher than that of SF graft. This provides adequate nutrition and oxygen support for the reconstruction of urothelial epithelium and the structural remodeling of smooth muscle cell bundles. Therefore, rapid epithelialization of grafts can further optimize urethral reconstruction and functional recovery.

## 4 EXPERIMENTAL SECTION

### 4.1 Materials and reagents

HA (Mw ≈ 180 kDa, MB7259‐1) was obtained from Meilunbio®. SF solution (Mw ≈ 100 kDa, 5154) was obtained from Sigma. Hexafluoroisopropanol (HFIP, 920‐66‐1) was purchased from Aladdin. Dulbecco's modified Eagle's medium (DMEM, 10567022), fetal bovine serum (FBS, 12483020), penicillin/streptomycin (P/S, 10378016), trypsin (0.25%, 15,050,057), phosphate‐buffered saline (PBS, pH 7.4, 10,010,023), Rhodamine‐ and Alexa Fluor‐488 conjugated phalloidin (R415 and A12379), DAPI (62248) were purchased from Gibco. Primary antibodies to cytokeratin‐5 (K5, ab75869), uroplakin‐3 (ab187646), α‐smooth muscle actin (α‐SMA, ab5694), CD31 (ab24590), and Ki67 (ab15580), and secondary antibodies for fluorescence staining were purchased from Abcam.

### 4.2 Preparation of electrospun SF and HA‐SF nanofiber

The HA solution was prepared by dissolving 200 mg HA in 10 ml HFIP and stirring until a uniform solution was obtained. A syringe with 10 ml HA solution was fixed on the precision injection pump. The flow rate of the electrospinning device was set at 1.5 ml/h, the distance between the electrospinning nozzle and the receiver (stainless steel with an outer diameter of ~2.7 mm or stainless steel rod of 10 mm) was set at 15 cm, while voltage was set at 12.3 kV. The speed of receiver was set at 1000 rpm, the temperature and humidity were 25°C and 45%, respectively. Electrospinning stopped after 2 or 6 h. A second syringe was loaded with 10 ml of fully mixed SF solution (50 mg/ml) and fixed to the precision injection pump. After connecting the electrospinning nozzle, the flow rate of the electrospinning device was set to 2.8 ml/h. The purpose of using a receiving device with 10 mm stainless steel rod was to obtain SF fiber film with HA‐coating on inner surface, while stainless steel with diameter of 2.7 mm was used to obtain tubular HA‐SF scaffold with inner diameter of 2.9 mm. HA‐coating SF nanofiber scaffold with an inner diameter of 2.7 mm and an outer diameter of 3.5 mm was obtained after 48 h electrospinning. The electrospinning setup of pure SF nanofiber film and tubular nanofiber scaffold in this study is consistent with the HA‐SF electrospinning procedure described above, but without the second HA electrospinning step.

The as‐prepared nanofiber films and tubular scaffolds were dried in a vacuum drying oven for 48 h to remove HFIP. The samples were then classified and immersed in the ethanol solution containing 50 mM 1‐ethyl‐3‐[3‐dimethylaminopropyl] carbodiimide hydrochloride (EDC) for 24 h, respectively. After crosslinking, the nanofiber samples were placed in a vacuum drying oven to remove organic solvents.

### 4.3 Characterization

The surface morphology and the diameter of the nanofibers were observed by SEM (SU8010, Hitachi) at an accelerating voltage of 5 kV.^45^The average diameter of nanofiber samples was measured from SEM images by ImageJ software. For each sample, 60 nanofibers were chosen randomly. The SF and HA‐coating SF nanofibers were characterized by FTIR spectra analysis with ATR head (ATR‐FTIR, thermo Nicolet, MA) in the range of 500–4000 cm^−1^ and scanning resolution of 2 cm^−1^.^46^, ^47^ The surface chemistry elemental was assessed by XPS spectroscopy by means of an XPS Kratos Axis Ultra HSA apparatus, which uses a micro‐focused monochromatic Al Kα X‐ray source (1486.6 eV) covering an analyzing area of 300 × 700 μm (900 W power).^48^ The TGA (Q50, TA Instruments) was performed to investigate the thermal stability behavior of different fibers to obtain the coating amount of HA on SF nanofiber surface in the range of 30–500°C with heating rate of 10°C/min under nitrogen atmosphere. The static water contact angles of different electrospun nanofibers were measured with an optical contact angle goniometer (VCA optima, AST, Inc.) in order to record the change of hydrophilicity. Static water contact angle data were recorded on four parallel samples after drying in a vacuum oven for 24 h and cutting into 24 mm^2^. The mechanical properties of electrospun nanofiber samples were measured and calculated by a tensile testing machine equipped with a 50 N load cell and a built‐in software (RTM502a; Shenzhen Vance Instruments Co., Ltd., China). The samples were cut into 1.1 × 6 × 0.5 cm^2^ and stretched at a constant rate of 5 mm/min.^48^ Four parallel tests were performed for each sample. The stiffness of HA‐SF and SF nanofibers was measured by AFM (MFP3DBio) in deionized water for 24 h, as described previously.^49^, ^50^ We used a standard sharp indenter model AC160 (Asylum Research) with averaged semi‐angle aperture = 19°. The elastic spring constant, k = 26 N/m, was calibrated in the air using thermal tune method. Young's modulus was determined by fitting force‐distance curve of the Sneddon model.

### 4.4 Cell growth and morphology on various nanofibers

The UCs of New Zealand rabbits were isolated using previously described methods,^11^, ^13^ and cultured in DMEM supplemented with FBS (10%, v/v), P/S (1%, v/v) in a 37 °C, 5% CO_2_ humidified incubator. After three passages, cells were seeded at a concentration of 10^6^ cells/cm^2^ on nanofibers and the medium was changed every day for the next 5 days. Prior to cell seeding, the nanofibers (diameter: φ = 24 mm) on TCPS of 6‐well plate were sterilized by ionizing radiation at 10 kGy from ^60^Co for 15 min.

At a predetermined time point of 96 h postseeding, the cell morphologies on the nanofibers were studied. The cell seeding nanofiber samples were washed twice with PBS to remove floating cells, and then fixed with 4% paraformaldehyde for 15 min at room temperature (RT). The fixed samples were washed three times with PBS, then dehydrated through an ethanol gradient (80%, 90%, 100%, 100%), and vacuum freeze‐dried over‐night, and conductive coating was implemented before SEM analysis, used to observe the status and morphology of cell growth on the nanofibers.

After 96 h of co‐culturing, the cell scaffolds were fixed according to the above steps, embedded with optimum cutting temperature compound (OTC, SAKURA, American) frozen embedding agent (4583, Solarbio) at −20°C, and finally sliced (20 μm) using constant cold box slicer at −22°C. Then the samples were stained with H&E staining kit (ab245880) according to the manufacturer instructions. The samples were visualized by pannoramic desktop microscope, with the representative areas were captured by Caseviewer software (version 2.3).

### 4.5 In vitro cell phenotype and proliferation study

After culturing for 24 and 120 h, the fixed cells seeding the nanofiber samples were permeabilized using 0.1% Triton X‐100 for 15 min and blocked with 2% serum albumin (BSA, B2064; Sigma‐Aldrich) for 30 min at RT. Then, the samples were incubated overnight at 4°C with primary antibodies uroplakin‐3 or Ki67 diluted 1:50 with PBS. Subsequently, after washing twice with PBS, the samples were incubated with Alexa Fluor‐488, or Cy3 conjugated anti‐mouse or anti‐rabbit immunoglobulin‐G (IgG) secondary antibodies diluted 1:400 with PBS for 30 min at RT. The F‐actin filaments of UCs were stained with Rhodamine‐ or Alexa Fluor‐488 conjugated phalloidin diluted 1:1000 with PBS for 30 min at RT, after washing twice with PBS the cell nuclei were stained with DAPI diluted 1:2000 with PBS for 10 min at RT. After washing three times with PBS, images were acquired using a Leica TCS SP5 CLSM. Uroplakin‐3+ and Ki67+ areas were shown as a percentage of the defined region area calculated using the ImageJ program (https://imagej.net/citing) considering 10 random fields per sample and *n* = 4 samples per group.

To confirm the Ki67 immunofluorescence results, CCK‐8 kit (ab228554, Abcam) was used to estimate the cell proliferation. We considered the cells seeding on six‐well plate TCPS as the positive control group. The cells were cultured with the same medium for 24 and 120 h, respectively. After medium replacement, the cells were washed three times with warm PBS, then filled with 100 μl CCK‐8 reagent and 900 μl serum‐free DMEM subsequently. After co‐culturing for 4 h, the supernatant was transferred into 96‐well plates and the absorption was measured with a microplate reader (Multiskan GO, Thermo Fisher) at 450 nm. Four parallel tests were performed for each sample.

### 4.6 Animal tests

All experimental procedures involving animals in this study were conducted under Institutional Guidelines for Animal Care and approved by the Animal Ethics Committee of Guangzhou Medical University (Guangdong, China). Male New Zealand rabbits aged 14 weeks were divided into two research groups: SF and HA‐SF scaffold graft group. Each animal received one graft, with nine animals in each group. The positive control was healthy urethral tissue, which was obtained from the 2 cm long urethral tissue cut during the manufacture of urethral trauma. As previously described,^11^, ^13^ under general anesthesia with pentobarbital sodium, the urethra skin was shaved and sterilized with Iodophor and 75% ethanol. Silk 5.0 fixing suture (A312; Jinhuan Medical Products Co., Ltd., China) was placed in the glans, a 6F catheter (Yiwu Medco Health Care Co., Ltd., China) was inserted into the bladder. A 2 cm skin incision was made at the proximal end of the glans, then the urethra within the cavernous tissue was separated. A 2 cm length urethral defect was made at 1 cm proximal to the base of the glans. A 2 cm length tubular SF or HA‐SF nanofiber (inner diameter of 2.7 mm and outer diameter of 3.5 mm) graft was fixed on the urinary catheter, and the native urethra and the graft were anastomosed end‐to‐end with PLGA 6.0 suture (LSP631; Jinhuan Medical Products Co., Ltd., China). Finally, the skin was sutured with PLGA 5.0 (LCC533). Postoperative daily analgesia of carprofen (Jinan Jinda Pharmaceutical Chemistry Co., Ltd., China) 4 mg/kg/day was used for 5 days.

Urothelial epithelial tissue (0.3 mm^2^) was isolated from the above‐mentioned healthy urethral tissue, while decellularized native urethral epithelial tissue was obtained by digestion with 0.2% collagenase IV (C2139, Sigma) at 37°C for 2 h (Figure S6).

As previously described,^11^, ^13^ under general anesthesia, the patency of the implanted graft was monitored by digital mobile X‐ray photography perspective system (SIEMENS, Luminos dRF) at the predetermined time point after implantation. Knowing that the graft is sutured 0.5 cm from the bottom of glans, and the length is 2 cm. The position of the graft can be determined on the X‐ray graph. Urine flow rate was obtained based on the amount of contrast agent (meglumine diatrizogamine, M861408; Guangzhou pharmaceutical Medicine Co., Ltd. China) discharged and time from the dynamic imaging system.

### 4.7 Histological and immunofluorescence assessment

At 6 weeks, and 14 weeks after urine flow rate experiment, the research animals were randomly sacrificed with an overdose of sodium pentobarbital (*n* = 3 animals per group), and the urethral tissue was dissected and separated. The retrieved graft samples were washed with normal saline to remove blood stains, fixed with 4% paraformaldehyde at RT for 24 h, dehydrated with gradient ethanol, embedded in paraffin, and cut into tissue sections (5 μm) were cut.^50^ Sample were then stained with Masson's trichrome (C1006, Servicebio), VVG (GP1035, Servicebio), and Sirius red (GP1033, Servicebio) according to the protocol of the reagent manufacturer. The sample visualization was carried out using Pannoramic DESK microscope, and the representative fields were obtained through Caseviewer software (version 2.3). Three slides per graft per time point were used for histological analysis. The average urethral epithelial thickness, the percentage of smooth muscle bundles and collagen were calculated from 10 random fields per slide using the imageJ software.^13^

For immunofluorescence staining of paraffin‐embedded tissue sections, the sections were first de‐paraffinized in xylene and then re‐hydrated in graded alcohol series, subjected to heat‐induced epitope retrieval in 10 mM citrate buffer (pH 6.0, G1201; Servicebio), followed by the immunofluorescence staining protocol described in Section 4.5. The average K5, Uroplakin‐3, CD31, or α‐SMA positive expression area was measured on 10 random fields of each slide (three animals in each group) with ImageJ software.

### 4.8 Statistical analysis

All experimental data were analyzed using GraphPad Prism (GraphPad Software, La Jolla) program. Where appropriate, a one‐way or two‐way analysis of variance (ANOVA) was performed to determine the significant difference with the Tukey post hoc test (*p* < 0.05). Unless otherwise stated, data and error bars are reported as mean ± standard deviation.

## 5 CONCLUSIONS

In conclusion, we have successfully prepared biomimetic tubular HA‐SF nanofibers by electrospinning and crosslinking process. The structure, morphology, and mechanical properties of the tubular HA‐SF nanofibers are close to the native urethral tissue of New Zealand rabbits. In vitro cell inoculation showed that the surface of hydrophilic HA‐SF nanofibers with biomimetic nano‐topography is more suitable for the growth of primary rabbit UCs. In vivo transplantation results showed that the dense gel coating at the inner wall of the tubular HA‐SF nanofibers can recruit endogenous UCs from the adjacent area occupying the defect site to form new urothelia tissue. The porous outer layer is conducive to more uniform infiltration of SMC, thus achieving tissue integration and optimal regeneration.

## AUTHOR CONTRIBUTIONS

**Yuqing Niu:** Conceptualization (equal); funding acquisition (supporting); investigation (equal); project administration (equal); resources (lead); writing – original draft (lead). **Massimiliano Galluzzi:** Investigation (supporting); writing – review and editing (lead). **Fuming Deng:** Investigation (equal); methodology (equal). **Zhang Zhao:** Investigation (equal); methodology (equal). **Ming Fu:** Investigation (equal); methodology (equal). **Liang Su:** Investigation (equal). **Weitang Sun:** Investigation (equal). **Wei Jia:** Investigation (equal). **Huiming Xia:** Conceptualization (equal); funding acquisition (lead); project administration (lead); supervision (lead).

## CONFLICTS OF INTEREST

All authors declared no potential conflicts of interest.

## Supporting information


**Appendix** **S1**: Supporting information

## Data Availability

All the associated data are available from the corresponding author upon reasonable request.
