# Supplementary material for: A biomimetic hyaluronic acid‐silk fibroin nanofiber scaffold promoting regeneration of transected urothelium
Source: Bioeng Transl Med. 2021 Nov 18;7(2):e10268. doi: 10.1002/btm2.10268 (PMC9115696; doi:10.1002/btm2.10268)
Supplement: Supplementary file 1 — Appendix S1: Supporting information [file BTM2-7-e10268-s001.docx]

**Electronic Supplementary Information (ESI)**

A biomimetic hyaluronic acid-silk fibroin nanofiber scaffold promoting regeneration of transected urothelium

Yuqing Niu^1^, Massimiliano Galluzzi^2^, Fuming Deng^1^, Zhang Zhao^1^, Ming Fu^1^, Liang Su^1^, Weitang Sun^1^,Wei Jia^1^, Huimin Xia ^1, *^

^1^ Department of Pediatric Surgery, Guangdong Provincial Key Laboratory of Research in Structural Birth Defect Disease, Guangzhou Women and Children's Medical Center, Guangzhou Medical University, Guangzhou 510623, Guangdong, P.R. China

^2^ Materials Interfaces Center, Shenzhen Institutes of Advanced Technology, Chinese Academy of Sciences, Shenzhen 518055, P.R. China.


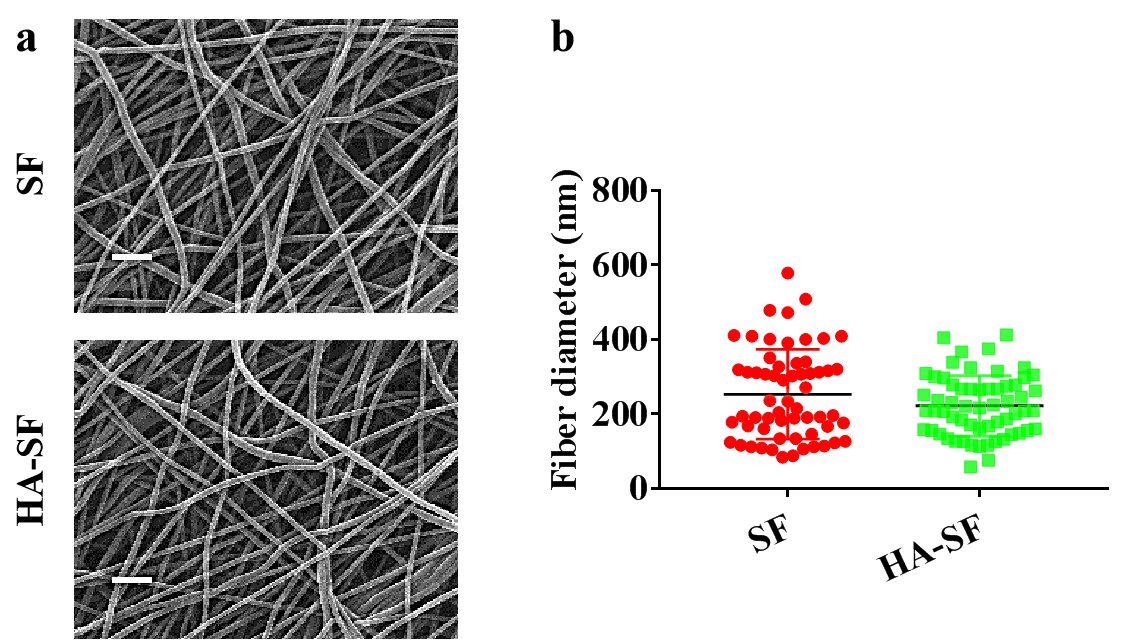


**Fig. S1. Morphology characterization.** (a) Representative scanning electron micrographs (SEM) of the inner surface of SF and HA-SF nanofiber before crosslinking. Scale bars, 1 μm. (b) Statistical data of nanofiber size of the above-mentioned nanofibers (*n*=60).


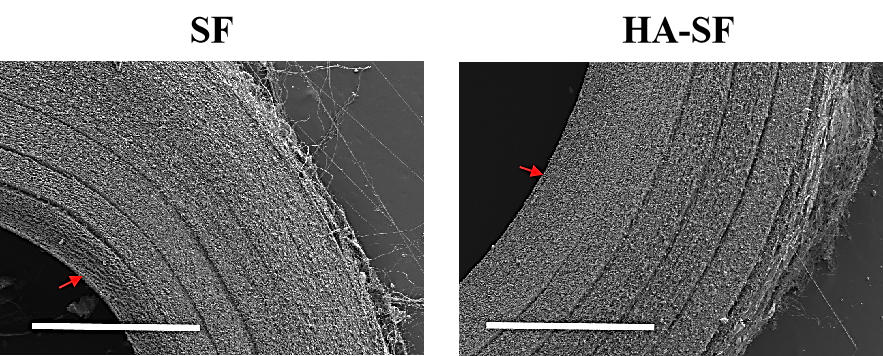


**Fig. S2. Representative** **SEM images of the cross-section of SF and HA-SF scaffold.** Scale bars, 500 μm. Red arrow indicates the inner surface of the tubular scaffold.

**Table S1. Summary of contact water angle, mechanical properties and decomposition temperature of SF and HA-SF nanofibers.**

| **Sample** | ***θ*H_2_O**  **(°)^a^** | ***E***  **(MPa)^b^** | ***δ***  **(MPa)^c^** | ***ε***  **(%)^d^** | ***E***  **(MPa)^e^** | **Td10**  **(℃)^f^** |
| --- | --- | --- | --- | --- | --- | --- |
| **SF** | **83**$\boldsymbol{\pm}$**1.6** | **1.3**$\boldsymbol{\pm}$**0.7** | **2.0**$\boldsymbol{\pm}$**0.2** | **407 $\pm$ 20** | **1.2** $\pm$**0.15** | **372.71** |
| **HA-SF** | **65.9**$\boldsymbol{\pm}$**1.2** | **0.83**$\boldsymbol{\pm}$**0.4** | **1.7**$\boldsymbol{\pm}$**0.4** | **387 $\pm$ 17** | **0.82**$\pm$**0.16** | **366.32** |

a: Contact water angle.
b: Young’s modulus from tensile test in dry state.
c: Stress at yield.
d: Strain at break.

e:Young’s modulus from AFM indentation test in wet state.
f; Decomposition temperature at 10% weight loss determined by TGA.

Sample abbreviation: SF means silk fibroin nanofiber film; HA-SF means hyaluronic acid-silk fibroin nanofiber film.


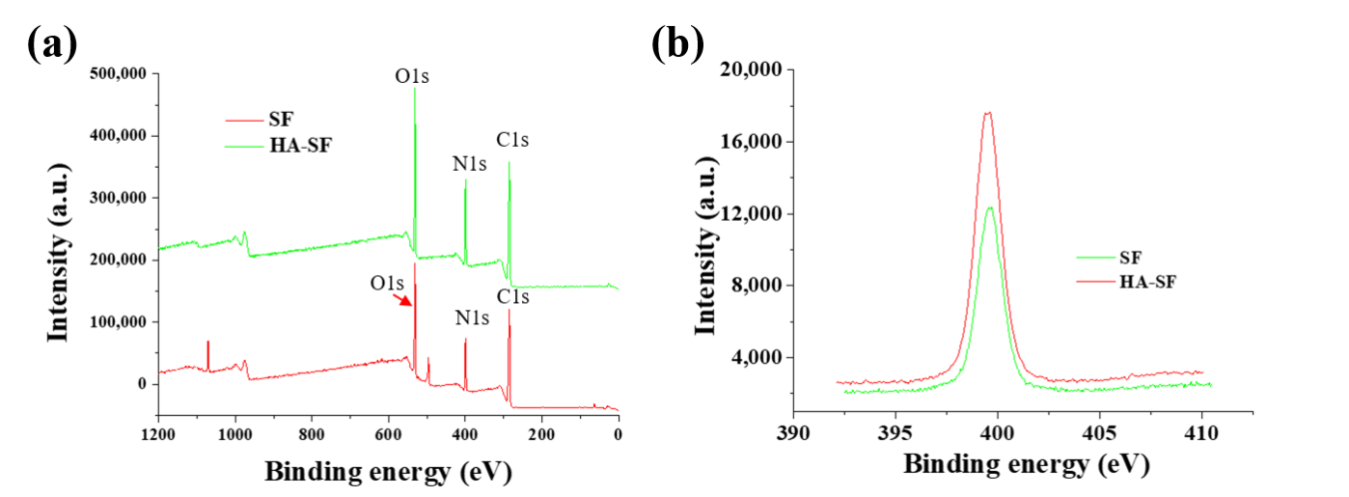


**Fig. S3. Surface chemical element distribution of the inner surface of SF and HA-SF nanofibers.**(a) XPS spectrum of O, N, and C peaks, (b) N1s curve fiting of high-resolution spectrum.


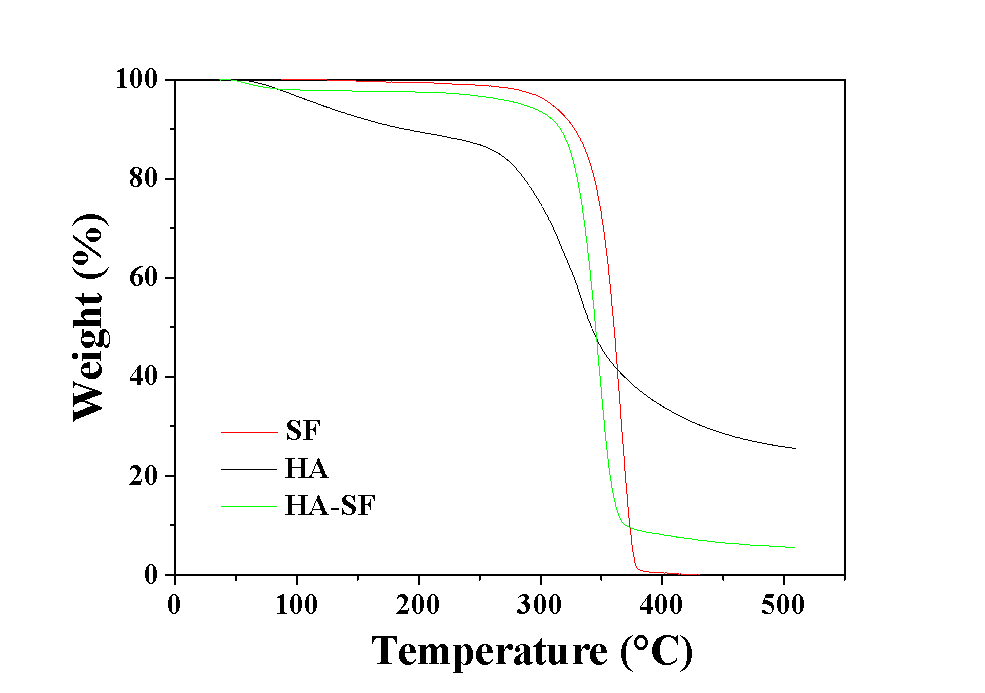


**Fig. S4. TGA thermograms of each tissue-engineered scaffold and HA.**


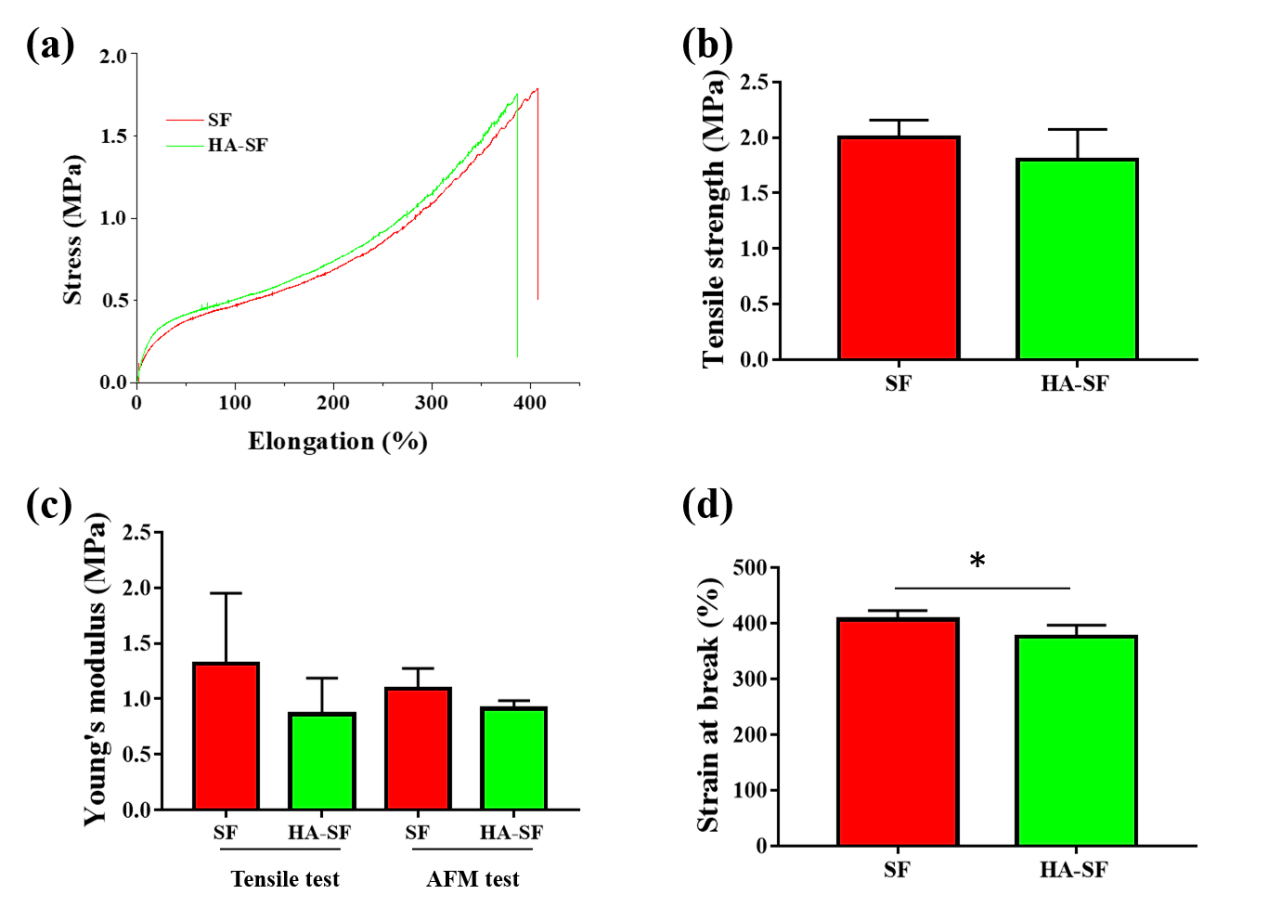


**Fig. S5. Mechanical properties of the electrospun nanofibers membranes.** (a) Typical stress-strain curves, (b) tensile strength, (c) Young’s modulus, and (d) strain at break. **p* <0.05

**
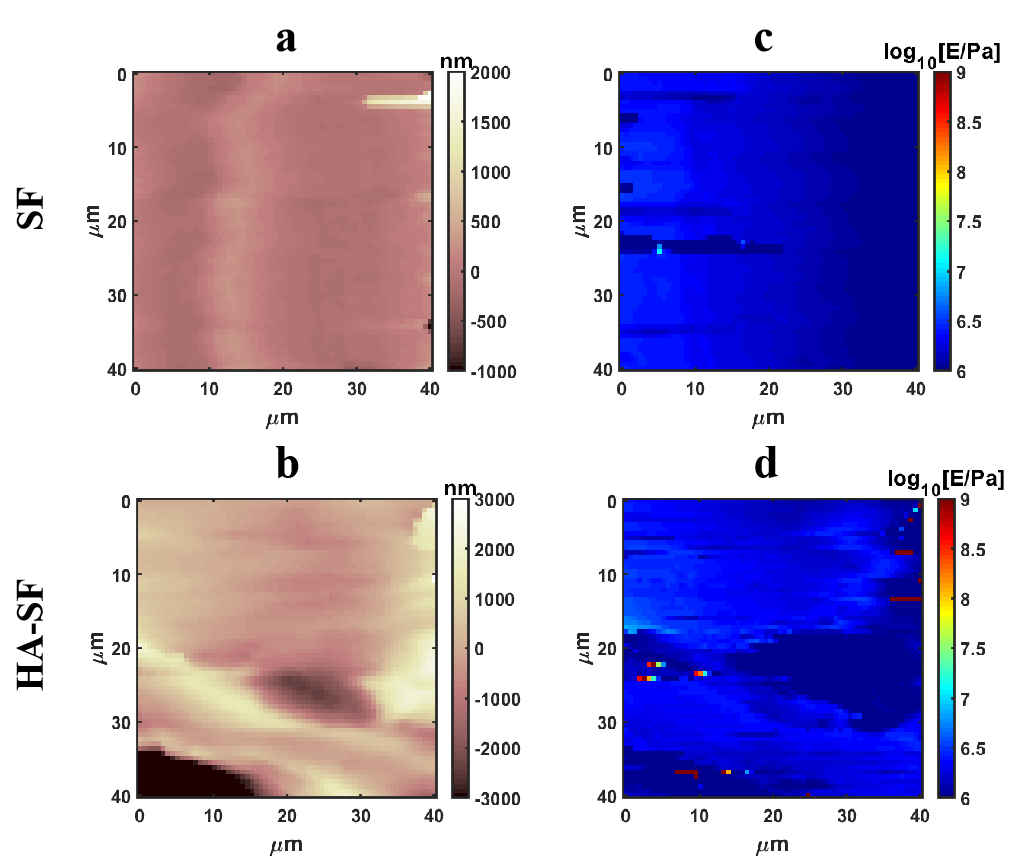
**

**Fig. S6. AFM nanomechanical measurments of nanofibers scaffolds.** High-resolution force volume (FV) (256$\times$256) compressed mophology (a) and Young’s modulus mechanical map (c) for SF nanofibers. Same order of images for HA-SF nanofibers (b, d).


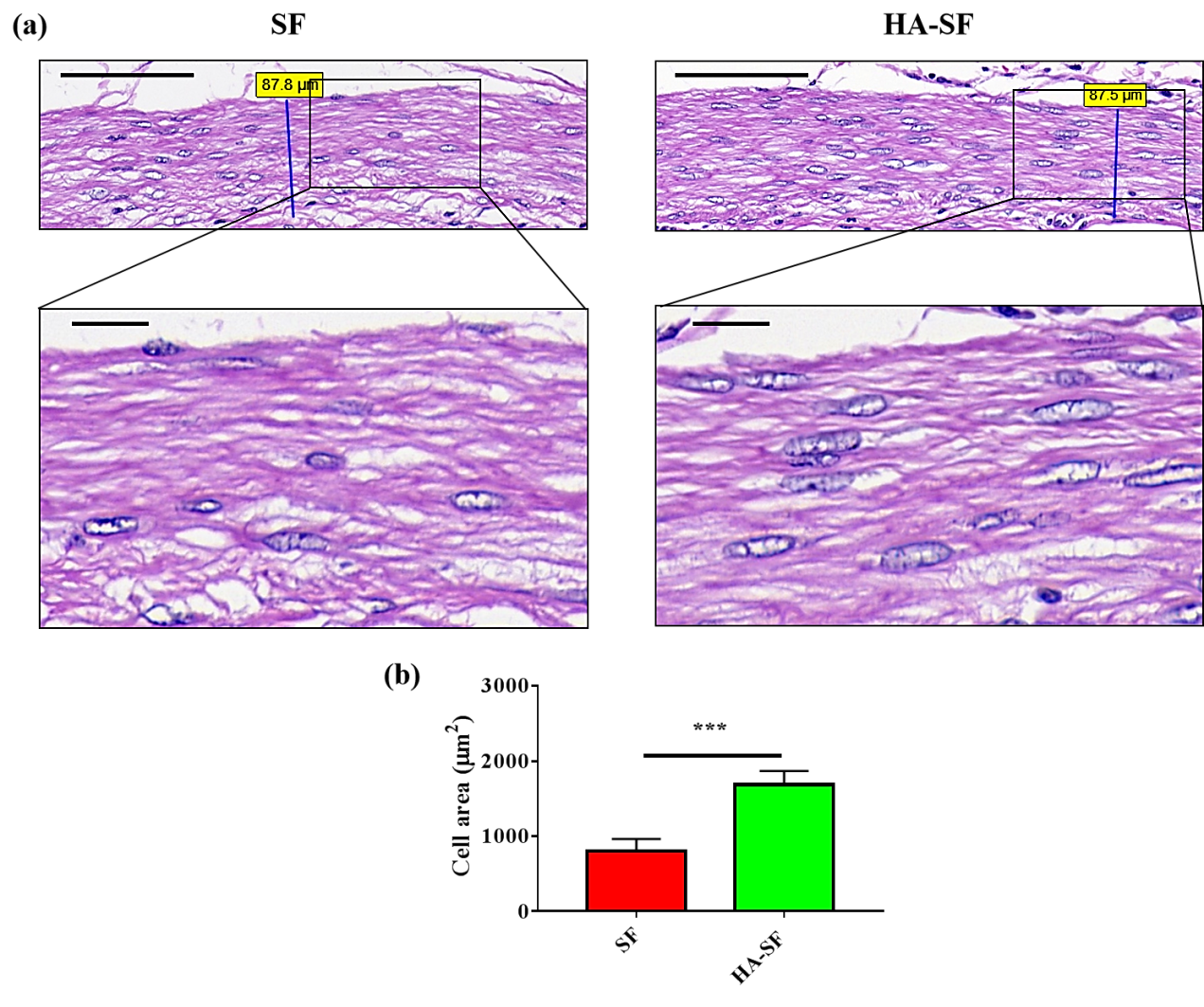


**Fig. S7. Growth behavior and morphological characteristics of primary UCs on SF and HA-SF nanofibers.** (a) H&E staining of the cross-section of cellularized SF and HA-SF nanofiber thin films. Scale bars, 100 μm (upper panel); 20 μm (lower panel). (b) Average area of primary UCs after 96 h post-seeding. ****p* < 0.01.


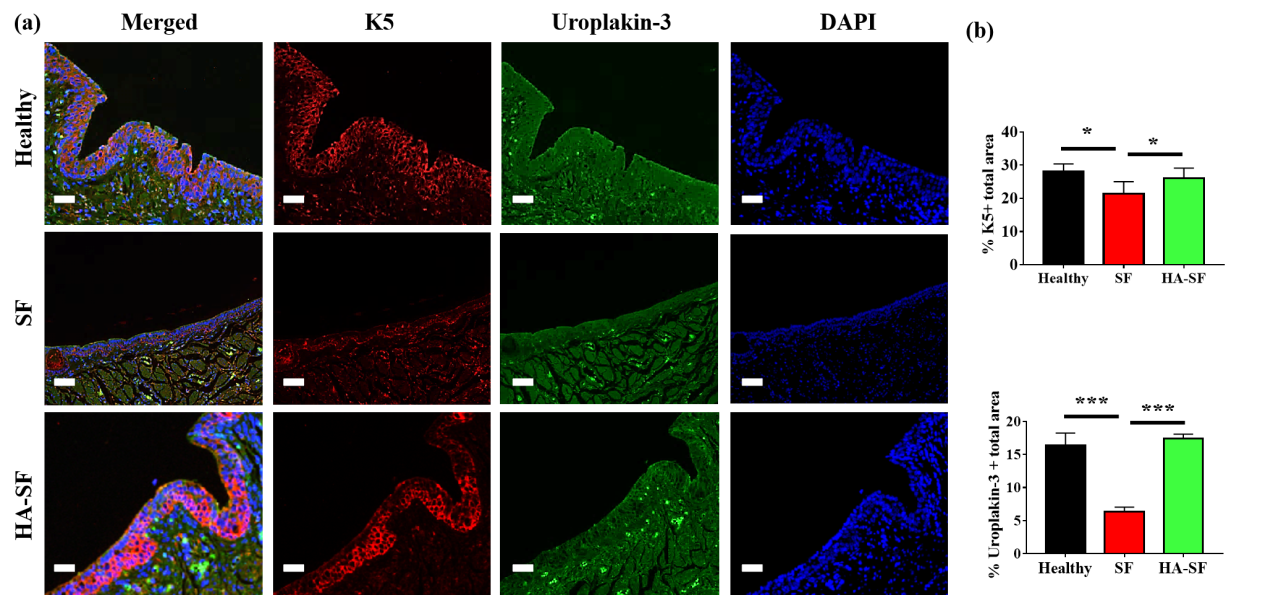


**Fig. S8. Immunofluorescence assessment**. (a) Representative CLSM images of the regenerated urethra epithelium. Blue indicates DAPI staining positive, red staining positive for basal layer progenitor cells, and green indicates positive staining for urethral plaque protein. Scale bars: 40 μm. (b) Statistical of the percentages of K5 positive and Uroplakin-3 positive in Fig. a (10 random fields per animal, *n*=3 animals in each group). **p* <0.05; ****p* < 0.01.


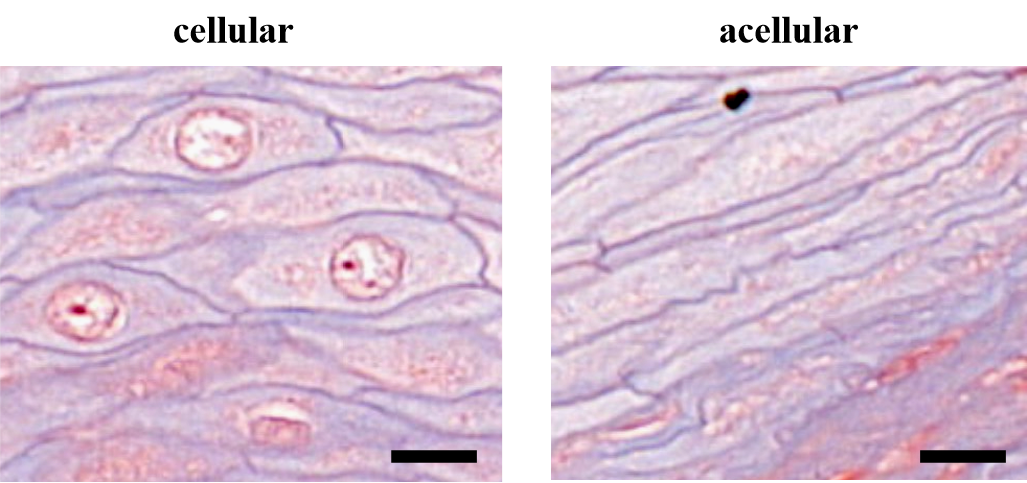


**Fig. S9. Masson's trichrome staining of the cross-section of native urethral tissue and decellularized urethral tissue**. Scale bars: 10 μm. After enzyme digestion, UCs disappeared, leaving fibrous ECM frame.
